# Pulmonary Delivery of an Ultra-Fine Oxytocin Dry Powder Formulation: Potential for Treatment of Postpartum Haemorrhage in Developing Countries

**DOI:** 10.1371/journal.pone.0082965

**Published:** 2013-12-23

**Authors:** Richard J. Prankerd, Tri-Hung Nguyen, Jibriil P. Ibrahim, Robert J. Bischof, Gemma C. Nassta, Livesey D. Olerile, Adrian S. Russell, Felix Meiser, Helena C. Parkington, Harold A. Coleman, David A. V. Morton, Michelle P. McIntosh

**Affiliations:** 1 Monash Institute of Pharmaceutical Sciences, Monash University, Parkville, Australia; 2 Biotechnology Research Laboratories, Department of Physiology, Monash University, Clayton, Australia; 3 Centre for Microscopy, Characterisation and Analysis, University of Western Australia, Crawley, Perth, Australia; 4 Department of Physiology, Monash University, Clayton, Australia; University of Strathclyde, United Kingdom

## Abstract

Oxytocin is recommended by the World Health Organisation as the most effective uterotonic for the prevention and treatment of postpartum haemorrhage. The requirement for parenteral administration by trained healthcare providers and the need for the drug solution to be maintained under cold-chain storage limit the use of oxytocin in the developing world. In this study, a spray-dried ultrafine formulation of oxytocin was developed with an optimal particle size diameter (1-5 µm) to facilitate aerosolised delivery via the lungs. A powder formulation of oxytocin, using mannitol, glycine and leucine as carriers, was prepared with a volume-based median particle diameter of 1.9 µm. Oxytocin content in the formulation was assayed using high-performance liquid chromatography-mass spectroscopy and was found to be unchanged after spray-drying. *Ex vivo* contractility studies utilising human and ovine uterine tissue indicated no difference in the bioactivity of oxytocin before and after spray-drying. Uterine electromyographic (EMG) activity in postpartum ewes following pulmonary (*in vivo*) administration of oxytocin closely mimicked that observed immediately postpartum (0-12 h following normal vaginal delivery of the lamb). In comparison to the intramuscular injection, pulmonary administration of an oxytocin dry powder formulation to postpartum ewes resulted in generally similar EMG responses, however a more rapid onset of uterine EMG activity was observed following pulmonary administration (129 ± 18 s) than intramuscular injection (275 ± 22 s). This is the first study to demonstrate the potential for oxytocin to elicit uterine activity after systemic absorption as an aerosolised powder from the lungs. Aerosolised oxytocin has the potential to provide a stable and easy to administer delivery system for effective prevention and treatment of postpartum haemorrhage in resource-poor settings in the developing world.

## Introduction

Postpartum haemorrhage (PPH), usually defined as the loss of >500 mL of blood within 24 hours of birth, occurs in more than 10% of all births and is most commonly attributed to uterine atony [1]. However, morbidity and mortality associated with PPH is seen almost exclusively in developing countries [2], where childbirth often occurs at home and the availability of trained healthcare providers and medical provisions can be minimal or inconsistent [2]. While current estimates attribute 35% of all maternal deaths to PPH, this statistic may be as high as 50% in some resource-poor settings [2]. Active management in the third stage of labour (AMTSL) comprising the administration of an uterotonic within one minute of birth, uterine massage and controlled cord traction are considered critical components for the prevention and treatment of PPH. Previous large scale clinical studies have demonstrated that when AMTSL was applied the incidence of PPH fell from ~18% to ~7%, when compared to expectant management alone [3,4]. 

Uterotonics including oxytocin, ergometrine and misoprostol are currently employed in AMTSL and are included in the World Health Organisation (WHO) list of essential medicines [5]. Of the three uterotonics listed, the WHO recommends the neuropeptide oxytocin, administered either intravenously (IV) or intramuscularly (IM), as the first line therapy for the prevention and treatment of PPH [1]. At present, oxytocin is only available as an aqueous solution for injection (5 IU or 10 IU, International Units) and is susceptible to degradation when cold-chain supply and storage is not maintained [6]. The alternate parenteral uterotonic, ergometrine, is also unstable at elevated temperatures and even more so when exposed to light [7,8]. Such cold-chain requirements present difficulties in developing countries where: (i) poor supply chain management, lack of infrastructure and local practices are often inadequate to maintain product stability [9]; (ii) many women give birth in rural facilities or at home where the supply of medical provisions, e.g., needle, syringes and alcohol swabs, required for injection are unavailable or inconsistent; and (iii) there are shortages of or limited access to skilled birth attendants or medical personnel to safely administer the injection [10]. 

Several approaches are being investigated to improve the availability and access to oxytocin in developing regions, including the development of single-use injection devices (Uniject®, Becton Dickinson) [11-13] and heat-stable, lyophilised powders that can be reconstituted immediately prior to administration [14]. However, these approaches do not obviate the requirement for either cold-chain supply and storage or skilled administration. Misoprostol, a prostaglandin analogue registered globally for the treatment of peptic ulcer disease, is a non-injectable uterotonic that, when administered either orally, sublingually or rectally, has demonstrated utility in the prevention and treatment of PPH. Programs are currently underway to distribute misoprostol as part of maternal health initiatives in developing countries [15]. However, current clinical evidence indicates misoprostol is less effective than oxytocin [10,16-18] and should be considered as a second-line uterotonic therapy, to be used in resource-poor settings only when oxytocin is unavailable [19]. Oxytocin, therefore, remains the first line uterotonic therapy for the prevention and treatment of PPH, and novel approaches are required to improve its access in resource-poor settings.

Administration of drugs via the respiratory tract is an effective route of delivery for some localised and systemic therapeutic treatments. For systemic drug exposure, achieved either via inhalation into the deep lung or deposition into the nasal cavity, the extensive vasculature and comparatively thin epithelium of these regions can facilitate effective absorption. Indeed, oxytocin has previously been developed (and in some countries continues to be registered) as a nasal spray for the augmentation of lactation [20], suggesting the respiratory system may provide an alternate route for systemic oxytocin delivery. As an aqueous solution, the oxytocin nasal spray encounters the same cold-chain requirements as the parenteral product. One alternative is to formulate oxytocin as a stable dry powder for inhalation (analogous to many current inhaled pharmaceutical formulations), with the potential to eliminate both the requirement for cold-chain supply and storage, and parenteral administration. Furthermore, a simple, easy-to-use inhalation or nasal delivery device may potentially reduce the reliance on skilled health workers to administer oxytocin (with the possibility for task shifting or self-administration) and eliminate the health risks associated with needle use in countries with endemic blood-borne diseases. For these reasons, the development and preclinical assessment of a needle-free, heat stable, dry powder inhalation product of oxytocin for PPH in resource-poor settings is investigated in this study. 

An ultrafine powder formulation of oxytocin was spray-dried and the physiological activity determined *ex vivo* using uterine (human and ovine) and tracheal (ovine) tissue. The *in vivo* activity of uterine smooth muscle was assessed electromyographically after pulmonary administration of oxytocin prepared as an ultrafine powder to postpartum ewes. The pharmacodynamic response was compared to an IM injection, the current recommended standard of care for PPH.

## Materials and Methods

### Preparation and characterisation of ultrafine powder oxytocin formulations

Two oxytocin formulations, containing either 10 IU (organ bath studies) or 200 IU (pulmonary delivery studies) of oxytocin, were prepared using the active pharmaceutical ingredient (API, > 99% purity) (Sunbow Biotech, Shenzhen, China) combined with a carrier mixture of mannitol, glycine and leucine (Sigma-Aldrich, St. Louis, MO, USA) in a 1:1:1 weight ratio (totalling 15 mg). The powders were prepared by spray-drying aqueous solutions of oxytocin with carrier to form ultrafine powders (1-5 µm), suitable for delivery to the broncho-alveolar region of the lung. A carrier-only formulation was also prepared as a negative control. Spray-drying was conducted in a Buchi 190 spray dryer (Buchi, Flawil, Switzerland) with the outlet temperature set at 70° C; air flow at 800 L/h and solution flow at 10 mL/min. Samples of the pre-spray-dried solutions and the resulting ultrafine dry powders were assayed for oxytocin content, to determine loss or degradation of the active ingredient during the spray-drying process (refer to LC-MS assay). 

The particle size of the prepared powders was characterised by laser diffraction using a Malvern Mastersizer 2000 fitted with a Scirocco 2000 measurement cell and a micro tray (Malvern Instruments, Worcestershire, UK). Measurements were performed at an air pressure of 3 bar; analyses used a refractive index of 1.5 and absorption of 0.01. The moisture content of each powder was determined with a 907 Titrando Karl Fischer titration unit (Metrohm AG, Herisau, Switzerland). The morphology of the spray-dried powder particles was examined using a Zeiss 1550 variable-pressure scanning electron microscope (Carl Zeiss, Oberkochen, Germany) at a magnification of x 5000.

### Liquid chromatography-mass spectroscopy (LC-MS) assay

Oxytocin concentrations throughout the study were measured using a validated LC-MS assay. The LC-MS system comprised a Shimadzu HPLC system coupled to a single quadrupole mass spectrometer and controlled by a CBM-20A system controller (Shimadzu Corporation, Kyoto, Japan). Chromatographic separation was made on a Gemini C18 column (50 x 2 mm, 3 μm) (Phenomenex Inc., CA, USA) connected to a SIL-20AHT autosampler and CTO-20A column oven maintained at 40°C. Analytical detection was performed using a LC-MS-2020 single quadrupole mass spectrometer with an electrospray ionisation probe in positive ionisation mode and with the capillary set to 4.5 kV. Desolvation gas was delivered (200 °C) at a rate of 1.5 L/min. Mobile phase was delivered as a binary gradient from a LC-20AD pump and comprised component A (95% ammonium formate buffer (0.5 mM) and 5% ACN) and B (95% ACN and 5% ammonium formate buffer (0.5 mM)) delivered as follows: 0-0.5 min: 5% B; 0.5-3.5 min: 5-80% B; 3.5-4 min: 80% B; 4-4.5 min: 80-5% B; 4.5-8.5 min: 5% B, over a 8.5 min run time. The flow rate was 0.3 mL/min with an injection volume of 5 μL. Shimadzu LC solution software was used for data acquisition and analysis. Oxytocin standards were prepared in the range of 0.1-12 IU/mL in water from a stock solution, with quality control standards at 1.5, 5 and 9 IU/mL incorporated into each LC-MS run. Assay accuracy and precision were ± 5% and ± 10% respectively at the lower limit of quantification (0.1 IU/mL).

### Ex vivo assessment of oxytocin bioactivity using isolated ovine and human smooth muscle tissue

The bioactivity of oxytocin present in the spray-dried powder was assessed by contractility studies using smooth muscle tissue excised from both the site of administration (distal trachea) and the site of therapeutic action (uterus). Isolated uterine and tracheal smooth muscle samples were obtained post-mortem from late-pregnant sheep. This study was approved by the ethics committee of the Royal Women's Hospital, Parkville, Australia (# 2012/33). Samples of human uterine smooth muscle were obtained at caesarean delivery and, prior to surgery, all participants gave informed written consent for collection of myometrial samples (5 × 5 × 10 mm) from the lower uterine segment in accordance with the Declaration of Helsinki.

The spray-dried powder formulation, equivalent to 10 IU of oxytocin, and oxytocin API alone were dissolved in water to form 1000 x stocks which were subsequently diluted in physiological saline solution (PSS) containing (mM): 120 NaCl, 5 KCl, 25 NaHCO_3_, 11 glucose, 1 KH_2_PO_4_, 1.2 MgSO_4_ and 2.5 CaCl_2_, continuously gassed with 95% O_2_ and 5% CO_2_. 

Two strips of uterine smooth muscle tissue, taken from each of the 8 women and 8 ewes, and ovine tracheal tissues (n=5) were tested. Each strip was suspended in an organ bath containing 20 mL of PSS at 37°C and connected to a Grass FT03C force transducer (Grass Instruments, Quincy, MA), which in turn connected to a Powerlab and LabChart Pro 7.3 hardware and software (ADInstruments, Sydney, Australia) for data collection and analysis. Tension was adjusted to 1 g over 1 h, with the PSS replaced every 20 min. Once a stable resting tension was established, the tissue was further rested until spontaneous rhythmic contractions developed. After 1 h the PSS was replaced with high K^+^ PSS for 5 min for uterine samples (HiK, normal PSS in which 100 mM NaCl was replaced with 100 mM KCl), and acetylcholine (ACh, 10^-5^M) for tracheal samples, in order to establish a standard contractile response. The organ bath was emptied and refilled with fresh PSS at 10 min intervals a further four times and rested for 45 min by which time spontaneous contractions had resumed. Cumulative additions of oxytocin stock solution were made to the organ bath at 10 min intervals to establish concentration-contraction curves over the range of 10^-11^ to 10^-7^ M oxytocin. Of the pair of tissues studied from each individual, one tested oxytocin API, while the other tested the spray-dried oxytocin formulation. In three separate experiments, the formulation alone, without oxytocin, was tested as a negative control.

### Pharmacodynamic evaluation of uterine contractile activity following pulmonary administration of oxytocin to postpartum ewes

A postpartum sheep model was established to assess the pharmacodynamic response of the postpartum uterus to oxytocin administered IM and via pulmonary routes. Studies were approved by the Monash University Animal Ethics Committee (approval # MARP-1-2011-037 and MARP-1-2012-035) and conducted in accordance with the Australian Code of Practice for the Care and Use of Animals for Scientific Purposes. 

Near-term merino ewes (day 140-145 of 150-155 day gestation, n=8, 40-60 kg) were anaesthetised using IV thiopentone (20 mg/kg; to facilitate placement of an endotracheal tube), and then maintained with isoflurane (2.5% in oxygen). The uterus was exposed via a midline abdominal incision (^~^7 cm in length), and three fine, insulated stainless-steel wire electrodes (Cooner Wire Co., Chatsworth, CA, USA) were surgically implanted into the muscle of the uterine wall, as previously described [21]. The wires were exteriorized through a small (^~^1 cm diameter) hole in the right flank of the ewe and the abdominal and flank incisions were sutured. A catheter was placed in the left jugular vein of the ewe to facilitate IV administration of dexamethasone for induction of labour. After 5 days of recovery, labour was induced in the ewes using 15 mg of dexamethasone (Intervet Australia, Victoria, Australia) administered IV as a bolus daily for 2 days. Lambs were delivered within 54 ± 4 h after the first dose of dexamethasone. Recordings of uterine electromyographic (EMG) activity were made continuously 24 h before and throughout delivery, and until the conclusion of the study. Oxytocin dosing commenced within 24 h post-parturition. A period of at least 1.5 h was left between dose administrations.

**Figure 1:**
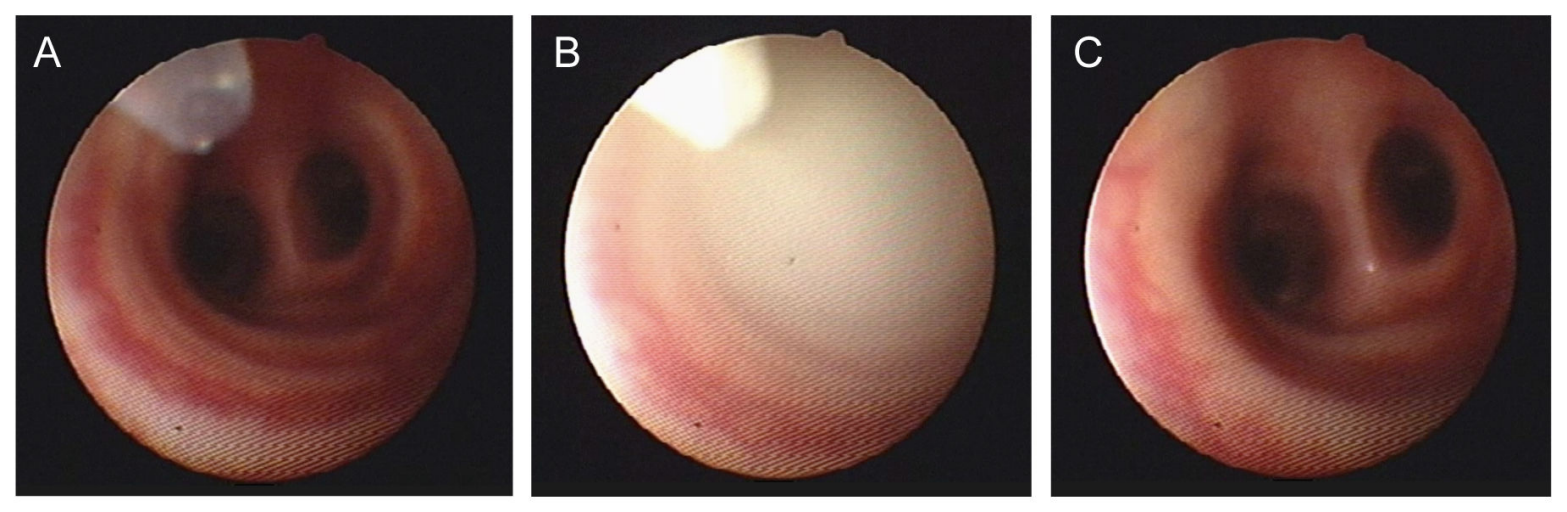
Administration of oxytocin powder formulation to the sheep lung. Screen images taken (A) prior to; (B) during and (C) 2 min after pulmonary administration of the oxytocin powder formulation above the first bronchial bifurcation.

Oxytocin was administered either as an aerosolised powder delivered to the distal end of the trachea (pulmonary), or by IM injection into the hind flank. For pulmonary administration, 15 mg of the spray-dried powder formulation containing 200 IU oxytocin was administered via the biopsy port of a fibre-optic endoscope (Model FG-16X, Pentax, New Jersey, USA) inserted via the nasal cavity in the conscious ewe. The endoscope was positioned at the distal end of the trachea, approximately 2-3 cm above the carina or first bronchial bifurcation (Figure 1). The oxytocin powder was delivered from a device comprising a DP-4 dry powder insufflator (with chamber extension) connected to an AP-1 air pump (Penn-Century Inc, Wyndmoor, PA, USA). To extend the device to the bronchial bifurcation, 130-140 cm of polyethylene tubing, 1.7 (OD) x 1.2 mm (ID), was attached to the end of the insufflator and fed through the endoscope to its distal tip. In a subset of sheep (n=3), ultrafine powder containing the same carrier excipients but no oxytocin was also administered *via* the pulmonary route as a placebo. In all cases, the delivered pulmonary dose was determined by weighing the device accurately before and after administration. For the IM dose, 10 IU of oxytocin was prepared in 0.5 mL of sterile saline and injected directly into the muscle of the hind leg. For all doses of oxytocin administered, EMG activity was recorded continuously before, during and for up to 2 h after dose administration or until baseline EMG activity of the uterus resumed.

### Data analysis

For the *ex vivo* bioactivity studies, the area under each 10 min contraction curve was determined for each uterine and tracheal strip in response to the increasing concentrations of oxytocin. The area under 10 min of spontaneous contractility was subtracted from the responses to oxytocin and the HiK contraction was designated as 100%. Sigmoid curves were then fitted to the oxytocin concentration*-*contraction data using the least squares method (GraphPad Prism, GraphPad Software, San Diego, CA, USA). Comparison of contractile activity was assessed using a one-way ANOVA of variance (GraphPad) with statistical significance accepted when *p*<0.05. 

Uterine EMG activity in the *in vivo* pharmacodynamic studies comprised bursts of spikes (indicative of uterine contractions [21]), which were analysed in terms of burst duration, bursts per 30 min and total duration of burst activity. In addition, the delay between the administration of oxytocin and the onset of the first observed EMG spike was also determined. Statistical comparisons between the treatments groups were assessed using a one-way ANOVA.

## Results

### Characterisation of spray-dried powders

**Figure 2:**
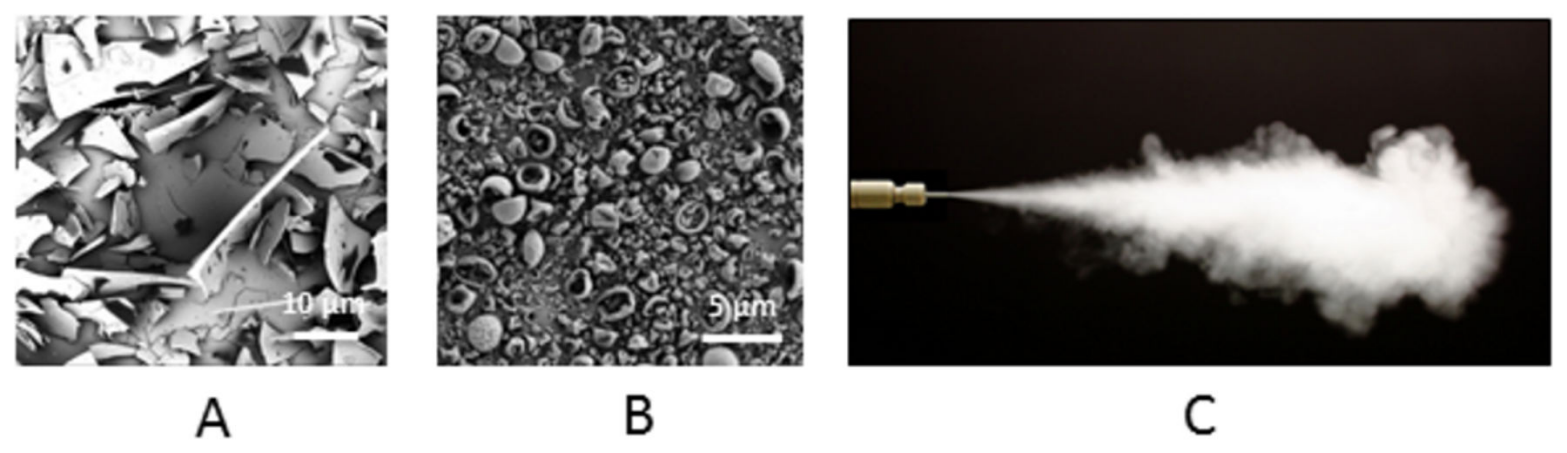
Images of oxytocin particles. Scanning electron micrographs of unprocessed oxytocin and oxytocin loaded carrier particles; (A) unprocessed commercial oxytocin (x 375); (B) oxytocin formulated as an ultrafine dry powder with the glycine-leucine-mannitol carrier (x 5000). (C) Well-dispersed aerosolised plume of ultrafine particles emitted from an insufflator.

The mean particle sizes of the three spray-dried powders (10 IU, 200 IU and placebo), as measured by laser diffraction, were similar, with volume-based median particle diameters ranging from 1.9 to 2.3 µm (Table 1). Similarities between the morphologies of the spray-dried powders, featuring spherical rugose microspheres (Figure 2B) were in marked contrast to the larger, angular fragmented appearance of unprocessed oxytocin (Figure 2A) when observed using scanning electron microscopy (SEM). The water content of the powders ranged from 0.23 - 0.26% w/w. When emitted from the Penn-Century insufflator, a well-dispersed aerosolised plume of the ultrafine powder was observed (Figure 2C). 

**Table 1:** Oxytocin ultra-fine dry powder formulation compositions and volume-based median particle diameters.

**Spray-dried Formulation**	**Glycine %**	**Mannitol %**	**Leucine %**	**Oxytocin %**	**Median Particle Diameter (µm)**
10 IU Powder	33.3	33.3	33.3	0.083	1.9
200 IU Powder	32.6	32.6	32.6	2.22	2.3
Placebo	33.3	33.3	3.33	-	2.3

Analysis of oxytocin content in the pre-spray-dried solution and resultant ultrafine powders indicated no statistically significant difference in oxytocin concentration (*p*>0.05), suggesting that degradation of oxytocin had not occurred during the spray-drying process. 

### Smooth muscle response to oxytocin using isolated uterine and tracheal tissue

**Figure 3:**
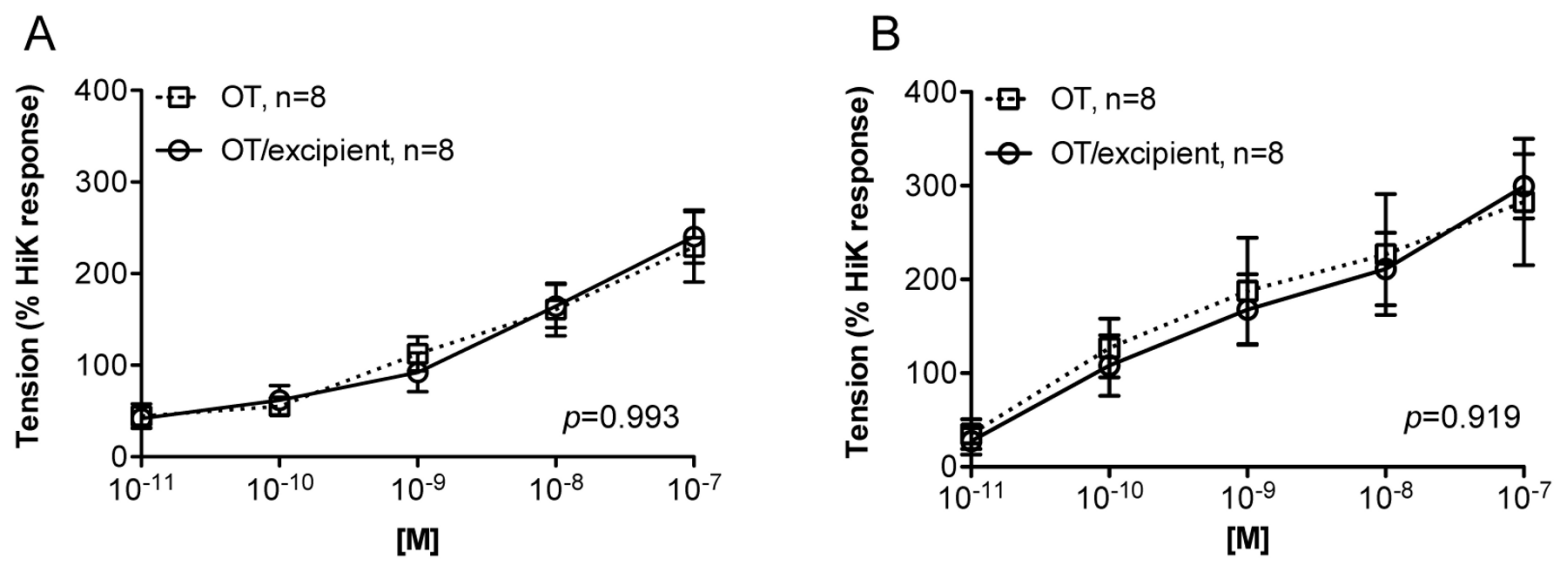
*Ex vivo* assessment of oxytocin induced uterine smooth muscle contractions. Effects of applied oxytocin on smooth muscle contractility *ex* *vivo* using (A) human (n=8) and (B) ovine (n=8) uterine tissue samples. Contraction was normalized as % of the response to high potassium (HiK) in PSS.

**Figure 4:**
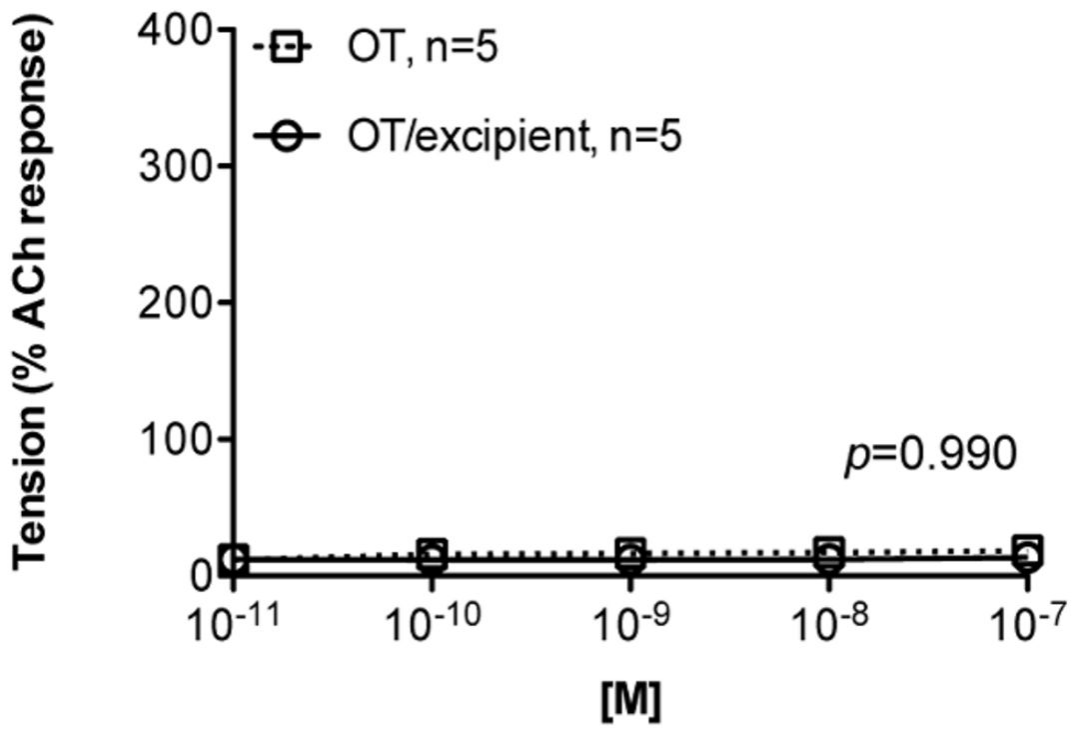
*Ex vivo* assessment of oxytocin induced tracheal smooth muscle contractions. Effects of applied oxytocin on smooth muscle contractility *ex* *vivo* using ovine (n=5) tracheal tissue samples. Contraction was normalized as % of the response to acetylcholine (ACh) in PSS.

Concentration-dependent increases in smooth muscle contractility occurred when excised human and ovine uterine muscle was exposed to oxytocin. There was no significant difference (*p*>0.05) in the contractile activity of human (Figure 3A) versus ovine (Figure 3B) uterine smooth muscle to cumulative increases in oxytocin alone or oxytocin reconstituted from the spray-dried powder formulation (*p*=0.99 for human, and *p*=0.92 for sheep, n=8), again demonstrating no loss in oxytocin potency after spray-drying. The placebo formulation had no effect on uterine contractility (data not shown). The magnitude of the contractile responses to oxytocin was the same in human as in ovine uterine smooth muscle strips (*p*=0.50). In contrast to uterine smooth muscle, no contractile response was observed across the oxytocin exposure range when ovine tracheal smooth muscle samples were prepared and assessed in the same manner (*p*=0.99, n=5) (Figure 4).

### Effect of pulmonary administration of oxytocin powder on uterine smooth muscle activity in vivo

Pulmonary administration of oxytocin ultrafine powder was well tolerated, with no sign of airway distress or irritability observed after dose administration. An average dose of 138 IU of oxytocin (range 100-190 IU) or 69% of the nominal dose, was emitted from the insufflator (Figure 2C) and delivered to the lungs of each postpartum ewe. 

**Figure 5:**
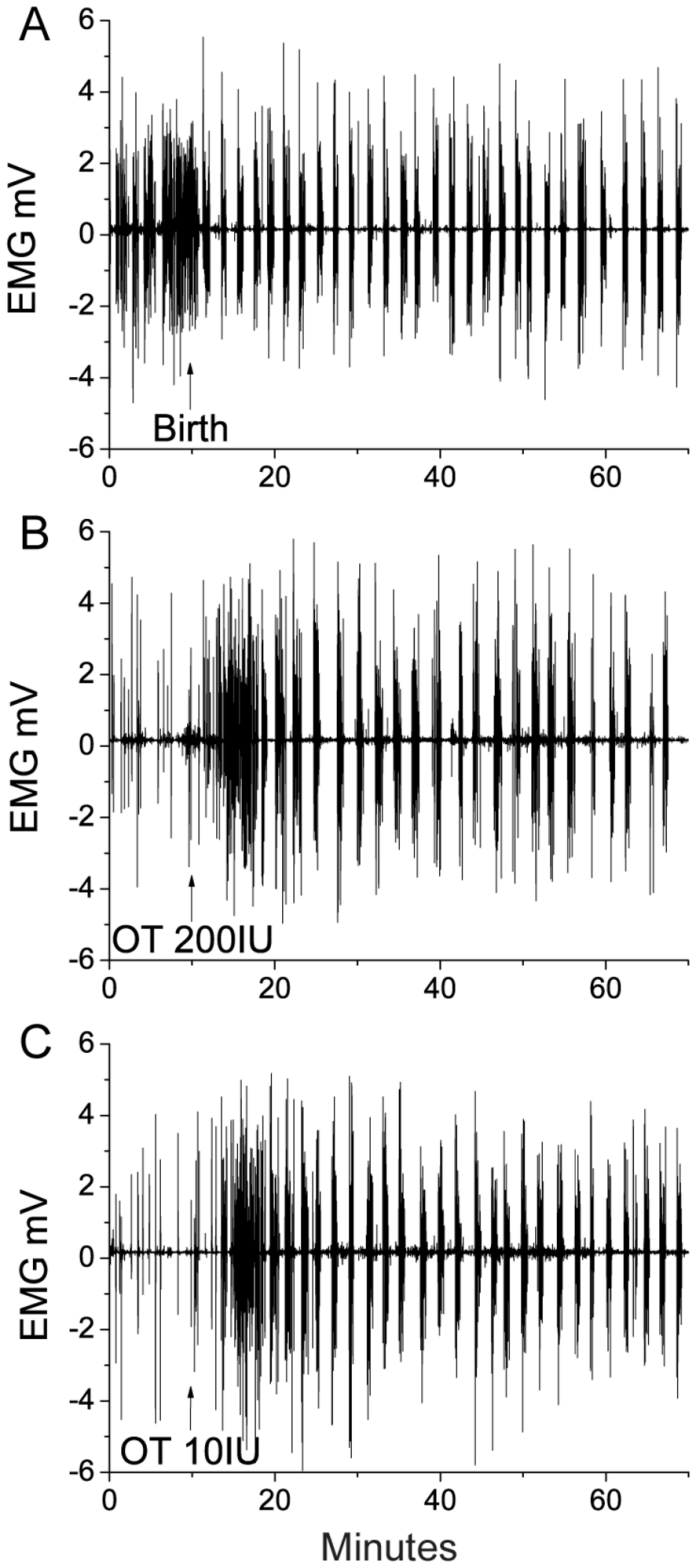
Representative electromyograph uterine activity over time in response to oxytocin administration. Representative electromyograph (EMG) activity over time (min) recorded *in* *vivo* from the uterine smooth muscle of sheep (n=8). (A) spontaneous activity during parturition and prior to oxytocin administration; (B) activity following pulmonary administration of 200 IU (nominal dose) of oxytocin prepared as an ultrafine spray-dried powder and; (C) activity following IM injection of 10 IU of oxytocin solution. Oxytocin dose and time of administration are indicated (OT = oxytocin).

EMG activity observed after pulmonary and IM oxytocin administration both closely mimicked the natural EMG activity occurring after parturition (Figure 5). The EMG responses that followed pulmonary administration (Figure 5B) were similar to those that occurred during the propulsive phase of delivery and for hours after delivery (Figure 5A). EMG activity in response to pulmonary administration also resembled that following IM administration of 10 IU oxytocin postpartum (Figure 5C).

**Figure 6:**
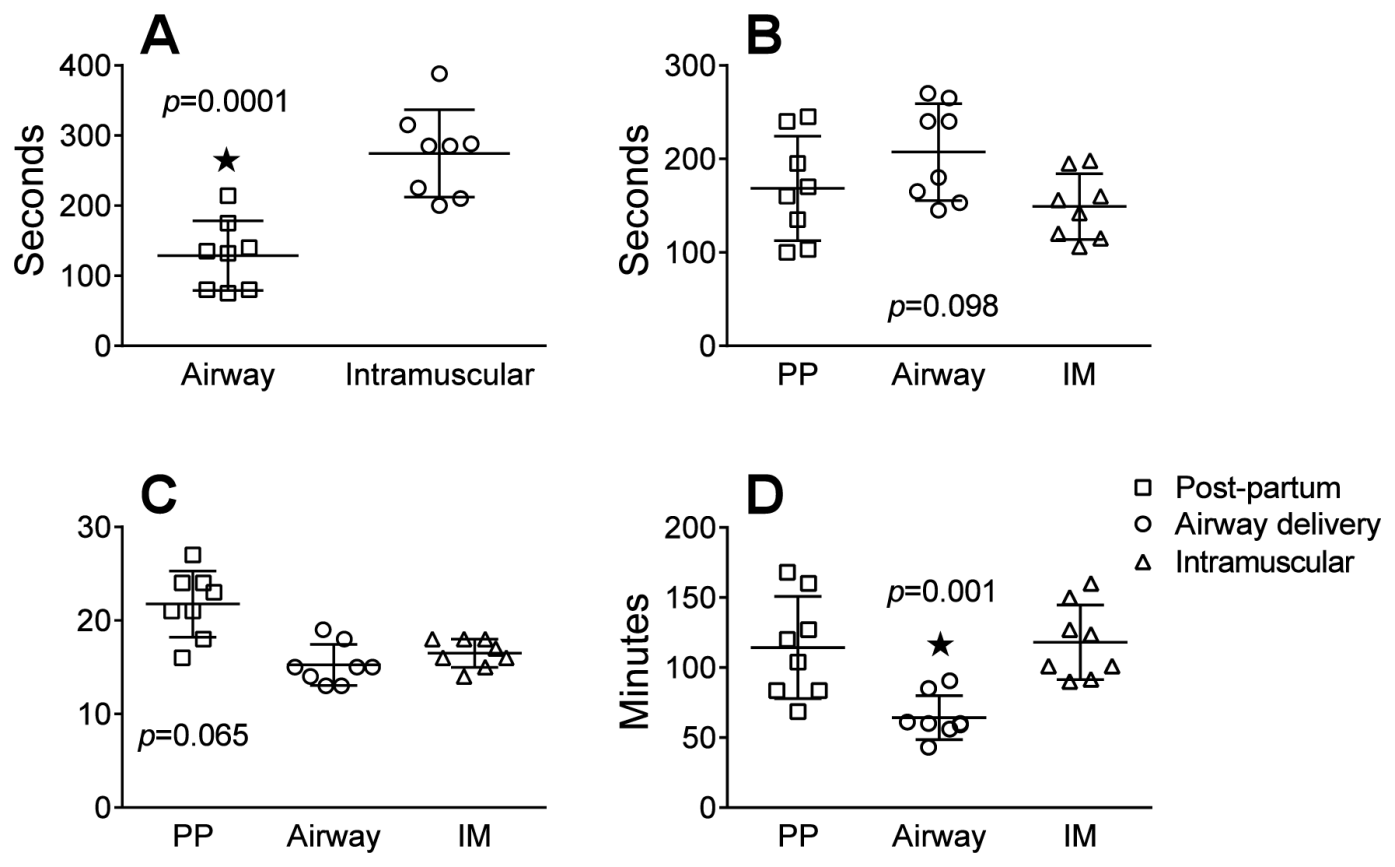
Parameters derived from electromyograph uterine activity in response to oxytocin administration. Electromyograph (EMG) parameters derived from group EMG profiles outlining; (A) time of onset; (B) duration of initial burst of activity; (C) number of activity bursts per 30 min and; (D) total duration of activity bursts. Data was collected immediately postpartum (PP) and after pulmonary (airway, 200 IU) or IM (10 IU) administration of oxytocin to ewes. Data presented as mean ± SD, n=8.

Results from the analysis of the EMG parameters (derived from the grouped EMG profiles) are presented in Figure 6. Pulmonary administration of oxytocin as a spray-dried powder resulted in a significantly faster onset of contractile response (129 ± 18 s, n=8) compared with the IM injection (275 ± 22 s, n=8; *p*<0.0001) (Figure 6A). There were no differences in the duration of the initial burst of contractions (Figure 6B) or the number of contractions over time (Figure 6C) when comparing the different modes of oxytocin exposure. However, the duration of EMG activity following IM administration of oxytocin (118 ± 9 min) was longer than that observed following administration via the pulmonary route (64 ± 6 min) (Figure 6D). The duration of IM oxytocin activity was similar to natural contractile activity observed postpartum (114 ± 13 min). The similarities in responses suggested that exogenous oxytocin delivered by either route would elicit a contractile response similar to that produced by endogenous oxytocin released during delivery of the lamb. Pulmonary administration of the placebo powder formulation did not elicit any EMG activity additional to the spontaneous contractions present prior to administration. This confirmed that the EMG response was attributable to the systemic absorption of oxytocin from the lungs. 

## Discussion

In this study, we investigated the capacity of a dry powder formulation of oxytocin to induce contractile responses in the uterine wall following pulmonary administration. Parallel studies performed *ex vivo* allowed comparisons between human and ovine uterine smooth muscle responses to oxytocin, while *in vivo* studies in postpartum sheep provided proof-of-concept data. Significantly, this is the first study to demonstrate that therapeutic levels of oxytocin can be systemically absorbed from the lung to stimulate a contractile response in the uterus analogous to that observed immediately after parturition. Currently, real-time uterine contractions cannot be continuously recorded in conscious animals or humans during the process of parturition. However, EMG activity can be recorded under these conditions and it is this electrical activity that strongly underpins (and is indicative of) contractions in sheep [22] and in women [23]. The sheep model was utilised not only due to the similarities in uterine physiology, but lung size, structure and physiology are also similar to human lungs, representing a relevant model system for human respiratory studies [24]. 

The lung has long been seen as an alternative route of administration for the systemic delivery of a range of bioactive molecules. Given that it is the only organ through which the entire cardiac output passes, potential exposure of the blood to a bioabsorbable drug is therefore high [25]. Effective absorption via the lung is facilitated by the high surface area (80-140 m^2^), extensive vascularisation and thin respiratory/alveolar epithelium (0.1-0.2 µm). These features are of particular relevance for peptide molecules, which normally require invasive parenteral administration to ensure effective administration. In order to penetrate the alveolar epithelium, polypeptides must not exceed 30 kDA [26]. In addition, the effective diameter of the formulated particles needs to be less than 5 µm to avoid impaction in the throat or upper airway after oral inhalation. In the present study, oxytocin was formulated as an ultrafine powder with a median aerodynamic particle diameter up to 2.3 µm, and at 1 kDa, oxytocin would be readily absorbed from the lung following pulmonary administration. ^125^I-labelled oxytocin had been previously measured in plasma for up to 3 hr after pulmonary instillation of a solution to the lungs of rats [27], suggesting that oxytocin is absorbed across the lung epithelium. In the present study we show that oxytocin administered via the pulmonary route is absorbed systemically, with detectable physiological activity seen at the site of the uterus.

The mechanisms governing the systemic absorption for oxytocin are not fully known. However, for macromolecules <40 kDa, absorption is thought to be facilitated primarily by paracellular diffusion or active transcytotic processes [28]. Using rat alveolar cell monolayers, vasopressin (1084 Da), a peptide structurally similar to oxytocin (1007 Da), has been demonstrated to readily diffuse across the membrane intact. Furthermore, peptidase activity was found to be localised mainly in the apical plasma epithelium and not in the alveolar space, where peptide degradation was considered minimal [29] and clearance rates were inversely related to molecular weight [30]. The latter point is consistent with significantly higher absorption of desmopressin (1069 Da) previously reported across rat [31] and pig [32] lung when compared to BSA (66 kDa). 

In addition to the relatively small size of the molecule (in comparison to the majority of therapeutic macromolecules), the suitability of oxytocin for pulmonary delivery may be further enhanced by co-spray drying of oxytocin with a mannitol, glycine and leucine carrier, or similar formulations. Co-formulation of peptides with sugars and amino acids is known to enhance the aerosolisation and fine particle fraction of powders (via modifications in particle morphology) [33] presenting the potential to optimize lung disposition, absorption and subsequent systemic activity. In addition, the maintenance of an amorphous glassy state enhances stability through inhibition of molecular mobility and interactions between peptide molecules [34]. Recent studies of several ultra-fine particle formulations, maintained at temperatures up to 50° C demonstrated that satisfactory stabilization could be achieved under extreme environmental conditions, similar to those where oxytocin would need to be administered in resource-poor areas, e.g., sub-Saharan Africa.

Oxytocin receptor expression in the myometrium is up-regulated during pregnancy in humans [35] and sheep [36], and therefore the response of the uterus to oxytocin (whether exogenous or endogenous) would be expected to be similar. The pharmacodynamic data showed that pulmonary administration of oxytocin mimicked the contractile response observed postpartum. Importantly, this provided both efficacy and physiological relevance for the pulmonary administration of the spray-dried oxytocin formulation in the postpartum period. Compared with the gold-standard intramuscular injection administered in the same ewes, the onset of contractions was significantly faster following pulmonary oxytocin administration (129 ± 18 vs. 275 ± 22 s). However, the duration of activity was significantly less (64± 6 vs. 118 ± 9 min). Nevertheless, the onset of electromyographic activity for both pulmonary and intramuscular administrations in sheep reflected the effective onset time in humans (2-3 min) when oxytocin is administered intramuscularly [37]. The onset time observed with inhaled oxytocin was faster compared with reported times for misoprostol after 400 µg was administered to women in the first trimester of pregnancy via oral (468 ± 102 s), vaginal (1164 ± 276 s) and sublingual (690 ± 276 s) routes [38]. The pharmacodynamic activity in response to the nominally higher inhaled dose reflected the lower bioavailability via the pulmonary route, estimated to be 10-20% for peptide drugs [39]. Since a large proportion of the formulation would be retained in the upper respiratory tract (and subject to mucociliary clearance and degradation by peptidases or proteases), the effect of the drug on the lung itself needs to be considered.

When exposed to increasing concentrations of oxytocin, excised ovine tracheal smooth muscle tissue exhibited no contractile response, suggesting that an inhaled dose of oxytocin would not induce bronchoconstriction. A previous study reported oxytocin receptor expression localised only in the vascular endothelial cells in normal human lung [36], an observation consistent with the bioactivity results observed with sheep tissue in the present study, and suggests acute delivery of oxytocin to the pulmonary region will probably not induce bronchoconstriction. Our *ex vivo* findings, together with the notable absence of airway distress or local irritation following pulmonary oxytocin administration, is supported by our preliminary *in vivo* observations that oxytocin delivered into the airways has no detrimental effect on airway function (Bischof et al, unpublished data). 

## Conclusions

Administration of oxytocin as an ultra-fine powder to the airways of postpartum sheep led to rapid systemic absorption, resulting in uterine electromyographic activity comparable to that induced by intramuscular injection, the current first line therapy for the prevention of PPH in resource-poor settings. Importantly, there were no contractile responses to oxytocin in isolated airway tissue, and no adverse effects observed following pulmonary administration in postpartum sheep. This is the first study to demonstrate that oxytocin can elicit uterine contractions after absorption as an aerosolised powder from the lungs, highlighting the potential for development of a safe, inexpensive and effective delivery system for the treatment of PPH in the developing world.
